# Development and Genetic Control of Plant Architecture and Biomass in the Panicoid Grass, Setaria

**DOI:** 10.1371/journal.pone.0151346

**Published:** 2016-03-17

**Authors:** Margarita Mauro-Herrera, Andrew N. Doust

**Affiliations:** Department of Plant Biology, Ecology, and Evolution, Oklahoma State University, Stillwater, OK 74078, United States of America; Pennsylvania State University, UNITED STATES

## Abstract

The architecture of a plant affects its ability to compete for light and to respond to environmental stresses, thus affecting overall fitness and productivity. Two components of architecture, branching and height, were studied in 182 F_7_ recombinant inbred lines (RILs) at the vegetative, flowering and mature developmental stages in the panicoid C_4_ model grass system, Setaria. The RIL population was derived from a cross between domesticated *S*. *italica* (foxtail millet) and its wild relative *S*. *viridis* (green foxtail). In both field and greenhouse trials the wild parent was taller initially, started branching earlier, and flowered earlier, while the domesticated parent was shorter initially, but flowered later, producing a robust tall plant architecture with more nodes and leaves on the main culm and few or no branches. Biomass was highly correlated with height of the plant and number of nodes on the main culm, and generally showed a negative relationship with branch number. However, several of the RILs with the highest biomass in both trials were significantly more branched than the domesticated parent of the cross. Quantitative trait loci (QTL) analyses indicate that both height and branching are controlled by multiple genetic regions, often with QTL for both traits colocalizing in the same genomic regions. Genomic positions of several QTL colocalize with QTL in syntenic regions in other species and contain genes known to control branching and height in sorghum, maize, and switchgrass. Included in these is the ortholog of the rice *SD-1* semi-dwarfing gene, which underlies one of the major Setaria height QTL. Understanding the relationships between height and branching patterns in Setaria, and their genetic control, is an important step to gaining a comprehensive knowledge of the development and genetic regulation of panicoid grass architecture.

## Introduction

Almost all plants branch, in order to develop an architecture that will enable them to capture and compete for resources. The ability of a plant to modify its architecture by differential branching and elongation is a characteristic that is determined both by developmental genetic programs and environmental influences, and the resulting form can affect a plant’s survival and reproduction [1, 2]. In plants selected for agricultural use, plant architecture is an important factor affecting yield (either grain/fruit or biomass) and suitability of a plant for agricultural purposes [3–10].

In grasses, there are two main types of branching, tillers that emerge from buds in the axils of nodes that are crowded together at the base of the plant, and aerial branches that originate from nodes above elongated internodes [11–17]. Tillers are produced by all wild grasses, and generally these tillers directly recapitulate the development of the main culm by producing adventitious roots that can eventually allow them to become semi- or fully independent plants [18]. Tillers can also be modified to facilitate horizontal spread by developing into rhizomes and stolons (below and above-ground prostrate stems) [19, 20]. Multiple cycles of tiller production may occur, leading to multiple orders of tillers on a plant. When aerial branches are present, they can initiate on the main culm and/or on tillers, and in some species, multiple orders of aerial branches may be produced.

Both tillers and aerial branches are the product of axillary meristems, but differences in their timing of appearance (onset of tiller production is earlier than that of aerial branches), and their partially separate genetic control as revealed by quantitative trait loci (QTL) mapping experiments in a variety of species [12, 17, 21–23], suggest that these two types of branches may best be analyzed separately. In addition, the distribution of these branching types across the grass family is not uniform, with species in some subfamilies, such as the Pooideae (including wheat, oats, barley and rye) rarely showing aerial branching, while most species in other subfamilies, such as the Panicoideae, generally exhibit aerial branching [24].

The genetic basis of plant branching and height have been extensively studied in the grasses by both forward and reverse genetic approaches [9, 14]. Branching is the result of complex interactions between environmental signals, gene regulatory networks, and hormonal pathways, and many genes have been implicated in both bud initiation and in elongation. Of these, initiation appears to be primarily under developmental genetic and hormonal control, while elongation is additionally affected by environment [14]. Major genes controlling bud initiation include *MONOCULM 1* (*MOC1*) in rice and *barrenstalk1* and *barren inflorescence2* (*ba1* and *bif2*) in maize [13, 16, 25]. Mutations of these genes suppress some or all axillary buds, including in the inflorescence.

Axillary meristems, once formed, are under developmental and environmental control, with many genetic and hormonal pathways converging on the TCP transcription factor *teosinte branched1* (*tb1*) in maize, and its homolog BRANCHED1 (BRC1) in Arabidopsis [26–28]. In grass systems studied to date, overexpression of this transcription factor results in repression of branch elongation, while the loss of expression results in the release of axillary buds to commence elongation [29–33]. Pathways that converge on *tb1* include those in the strigalactone pathway such as *D3*, *HTD1*/*D17* and *D10*, which are rice orthologs of the Arabidopsis MORE AXILLARY BRANCHING genes MAX2, MAX3 and MAX4, and the Pisum RAMOSUS genes RMS4, RMS5 and RMS1, respectively [34–39]. Additional genes influencing axillary meristem elongation include auxin efflux carrier genes such as *OsPIN1b* [40] and *OsPIN2* [41]), miRNAs that regulate auxin receptors such as the *OsmiR393* family [42], ISOPENTENYL TRANSFERASE (IPT) genes involved in regulating cytokinin levels [43], genes involved in gibberellin catabolism, like C_20_ GA 2-oxidases (GA2ox) [44], and genes regulating brassinosteroid biosynthesis [45, 46].

The amount of branching that a plant produces is only one component of plant architecture, and the relative elongation of those branches versus the height of the main culm contributes greatly to the eventual form of the plant. The genetic regulation of height has been the focus of much research, because it is highly correlated with plant yield and flowering time [9]. Elongation and branching are often related phenomena, and changes in height may be correlated with changes in branch number. For example, the hormone gibberellin induces elongation and has been shown to simultaneously upregulate *OsTB1* expression to inhibit tillering in rice [44]. Opposite changes are seen with many of the dwarf mutants in the strigalactone pathway in rice, which are characterized by an accompanying increase in branching [36–39].

The effect of plant architecture on biomass has been studied in sorghum, maize, and switchgrass, and in each case a positive relationship has been observed between biomass and height [47–52]. Biomass is also often correlated with length of time to flowering, this being correlated with the eventual height of the plant [51]. In grasses, the relationship between time to flowering and biomass is particularly close, as inflorescences terminate the branch, eliminating the possibility of further leaf production on a branch after flowering. A positive effect of branch number on biomass has been observed in switchgrass and sorghum [17, 47], suggesting that manipulation of both height and branching are possible methods to achieve higher biomass.

The developmental genetic regulation of height and branching in panicoid grasses such as sorghum, maize, switchgrass, and millets is less well understood than in rice, possibly because the strong suppression of branching in most panicoid crops makes its investigation more difficult. To aid in understanding the development and genetic control of plant architecture in panicoid grasses, we have chosen to use the Setaria model system, which combines a developmentally plastic wild ancestor (*S*. *viridis*, green foxtail) with a domesticated and more developmentally canalized descendant (*S*. *italica*, foxtail millet) [53–56]. These grasses are in the same subfamily as maize and sorghum (Panicoideae) but in a different tribe (Paniceae). Tribe Paniceae also includes switchgrass (*Panicum virgatum*) and pearl millet (*Pennisetum glaucum*). The Paniceae diverged approximately 27–28 mya from the tribe Andropogoneae, containing maize and sorghum [57, 58]. This contrasts with a distance between both subtribes (subfamily Panicoideae) and subfamily Ehrhartoideae (containing rice) of approximately 50–65 mya [57, 59].

**Fig 1:**
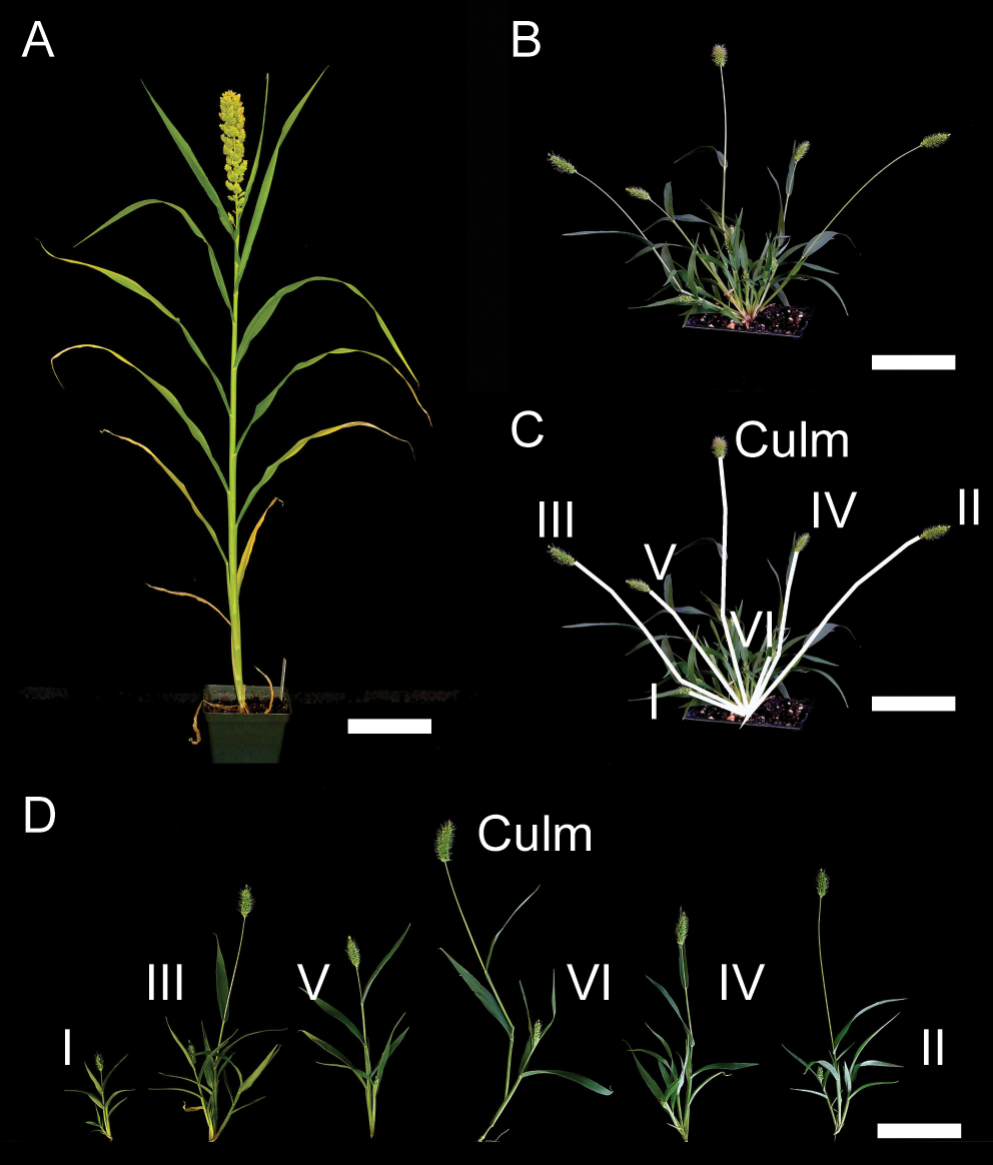
Growth form of *Setaria italica* (foxtail millet) and *S*. *viridis* (green foxtail). *Setaria viridis* produces multiple tillers and aerial branches whereas *S*. *italica* only produces one or a few tillers and no aerial branches. A. *S*. *italica*, B. *S*. *viridis*, C. *S*. *viridis* showing tillers numbered from the first produced tiller (I-VI), D. *S*. *viridis*, with tillers separated so that secondary tillers can be better displayed. Scale A = 12 mm, B-D = 8 mm.

*Setaria viridis* is much branched and very variable in morphology whereas *S*. *italica* shows a typical domestication phenotype of increased height of the main culm with additional nodes and leaves, large inflorescences, and suppression of tillers (Fig 1). Preliminary QTL mapping of branching traits in an F_3_ family derived from a cross between *S*. *italica* and *S*. *viridis* suggested that there was significant heritable variation in branching [21, 22]. The study reported here uses F_7_ recombinant inbred lines (RILs) derived from the same cross, together with a recently developed genetic map [60], to study the relationship between branching, height, and biomass in field and greenhouse growth conditions, and to investigate genetic regions controlling these traits at vegetative, first flowering, and harvest stages. The use of measurements at multiple time-points is advantageous as the timing and spatial location of branching events is distributed over the growth cycle of the plant. In fact, QTL analyses of tiller production and elongation in other species such as switchgrass, rice and wheat have identified different quantitative trait loci at different stages of the lifecycle [48, 51, 61–65]. As the Setaria system is phylogenetically close to potential biofuel grasses such as switchgrass, napier grass and pearl millet, understanding the relationships between the components of plant architecture across multiple developmental stages may aid in the breeding of better plant architectures for a variety of agricultural purposes including biomass and grain production.

## Materials and Methods

### Plant materials and experimental design

An F_7_ recombinant inbred line (RIL) population comprising 182 lines derived from an interspecific cross between *S*. *italica* (accession *B100*) and its wild relative *S*. *viridis* (accession *A10*) [66] was evaluated in two separate trials for plant architectural traits at vegetative, flowering, and harvest stages. The greenhouse trial was conducted at Oklahoma State University during the months of mid-June to mid-October in the summer of 2008, while the field trial was conducted at the Cimarron Valley Field Research Station in Perkins, Oklahoma during the months of May to July of 2010. In both trials seed was planted in the greenhouse in MetroMix^®^ 360 growth medium (Sun Gro Horticulture, Canada). For the greenhouse trial, seeds were planted in 12 x 12 cm square pots and kept in these pots for the remainder of the trial. In the field trial, seed was germinated in a greenhouse in 3 x 3 cm square pots and seedlings were transplanted to the field 12 days after sowing, at approximately the two-leaf stage. These two trials varied in growing conditions, including photoperiod (greenhouse trial was planted almost six weeks later in the year than the field trial), spacing, and environmental variables such as temperature, light intensity, and humidity. Light intensity in the greenhouse was approximately 2/3 that of field conditions, and plants were grown without supplemental lighting. For detailed growing conditions refer to Mauro-Herrera et al. (2013).

Both trials were conducted under a Randomized Complete Block Design (RCBD). The greenhouse trial consisted of six blocks with one replicate per genotype per block. The field trial had two blocks with three replicates per genotype per block. Watering for both trials was provided as needed, but plants in the field were fertilized with nitrogen only at the beginning of the experiment whereas the greenhouse plants were fertilized with dilute fertilizer Jack’s 20-20-20 (Jr Peters Inc.) 1/15 strength as they were watered each day.

### Phenotypic measurement and analysis

Phenotypic measurements were done at the vegetative, flowering and maturity (harvest) stages of the plant life cycle. Measurements at the vegetative stage were done at a single time point for each trial (14 days after planting for greenhouse, 11 days after planting for field), and measurements for field trial were done before the transplantation of the seedlings to the field. The vegetative stage was measured before any of the RILs had started to initiate an inflorescence meristem. Because measurements at the vegetative stage were done at a single time point they incorporate both developmental variation in growth rate as well as variation in germination time (seeds generally took four to five days to germinate but several took six or even seven days). Flowering is defined as the first appearance of the inflorescence as it emerges from the flag leaf sheath, and is thus a specific developmental time point in all RILs, even though the RIL lines varied in growth rates. In the field trial plants were harvested when the main inflorescence had over 50% mature seed (defined as a change in color from green to brown), whereas in the greenhouse trial, plants were left to grow until most of the inflorescences had mature seed. Even though the criterion for harvesting differed between the two trials, plants were still harvested in their order of maturation. All measurements at maturity were done on harvested plants. Height and tiller number were measured at the vegetative stage, height, tiller number and aerial branch number were measured at the flowering stage, and height of the main stem (culm), height of the tallest tiller, node number, tiller number, aerial branch number, and total biomass were measured at the harvest stage.

Culm height was measured from the base of the plant to the highest leaf collar of the youngest leaf on the main culm. Height of the tallest tiller was measured from the base of the plant to the highest leaf collar of the youngest leaf on that tiller. Both culm and tallest tiller height were recorded because there can be great variation between these, leading to quite different architectures and different potential for biomass accumulation over the life of the plant.

Node number was recorded as the total number of nodes visible along the main culm (but does not include the cluster of basal nodes whose internodes do not expand to separate them). Tiller number was scored as all those branches originating from nodes at the base of the main stem/culm (with unexpanded internodes), and included all orders of tillers, comprising primary (tillers originating from the main stem) and tillers of higher orders (tillers originating from other tillers). Aerial branch number includes all those branches originating from an aerial node, either on the main stem, on a tiller or on another aerial branch. We also analyzed total branch number, the sum of both tillers and aerial branches on a plant. Total dry-weight above-ground biomass was measured by weighing plants with the roots removed, and which had been dried in an oven for at least one to two weeks. In order to determine if there were any differences in the amount of biomass produced on a daily basis, given the variation in days to harvesting in both trials, an average biomass growth rate value was estimated as total biomass divided by days to harvest.

#### Data transformation

Distributions of the least square means for every trait across the RIL population were examined for normality using normal Q-Q plots [67]. Traits with deviations from normality were transformed as necessary, and transformed data used in subsequent analyses. Square root transformed traits included height, tiller number, aerial branch number, and total branch number at flowering, and tallest tiller height, total biomass and biomass growth rate at harvest. Natural log transformed traits included height at the vegetative stage and node number at harvest. Box-Cox transformed traits included tiller number at the vegetative stage, and tiller and aerial branch number at harvest.

#### Phenotypic correlations and PCA analysis

Relationships between traits were explored by non-parametric bivariate two-tailed Spearman phenotypic correlations at all stages, and between biomass and trait values at different stages of growth. Principal component analyses (PCAs) were performed on transformed trait data at flowering time and at harvest. Component axes rotation in the PCAs was performed with the varimax procedure, and scree plots used to select components that explained significant proportions of the variance. All analyses were performed with SPSS version 21 (IBM SPSS, Armonk, NY).

### QTL analyses

For QTL analyses we used the previously published 684 marker genetic map, that identified nine linkage groups corresponding to the nine chromosomes of Setaria [60], and the transformed trait dataset. QTL Cartographer Unix version 1.16 [68, 69] was used for QTL analyses with the composite interval mapping (CIM) method, a genome scan interval of 1 cM, a window size of 10, and the forward and backward regression method [68–71]. LOD threshold values were estimated via 1000 permutations [72, 73]. QTL regions were defined based on co-localization of QTL for traits occurring in more than one trial or developmental stage.

QTL regions identified in Setaria were compared with previous studies in Setaria and with QTL found in other grass species by establishing the genomic coordinates of each QTL from published reports and comparing their genome position with that of the Setaria QTL using the SynMap module in CoGE [74, 75].

#### Epistasis

We used Epistacy [76] to compute epistatic interactions. Epistasis was calculated for all pairs of markers, giving 233,586 tests for significance for each trait. Using the eigenvalue variance method to correct for linked markers [77] gives 222,826 tests, so that a Bonferroni adjustment to the experiment-wide error rate of P < 0.05 gave an individual test P-value of P < 2.3 x 10^−7^ [78].

#### Candidate gene analysis

QTL regions for plant architecture and biomass traits that were consistently identified in both trials and/or across developmental stages were searched for candidate genes. Sequences of candidate genes were obtained for genes identified in primary literature and review papers on plant architecture. In addition, sequences of candidate genes from well-studied species were obtained by searching PubMed-NCBI and Nucleotide-NCBI with the query words “green plants” and “tiller gene” or “green plants” and “height gene”. In both searches sequences of genes were only downloaded when indication of a function in plant architecture was reported in the cited publication. A BLAST analysis was conducted with downloaded gene sequences against the current *Setaria italica* genome assembly (version 10 available on www.phytozome.jgi.doe.gov) to identify the gene and its position on the genome. A reciprocal blast search in NCBI or TAIR was done with top and second-best *Setaria italica* BLAST DNA sequence hits for genes in the QTL regions of interest to validate that the identified Setaria sequence corresponded to the gene identified in other species.

## Results

### Phenotypic variation

All values are given as mean ± standard deviation.

#### Vegetative stage

At the vegetative stage there was substantial variation in plant height amongst all genotypes, reflecting differences in growth rate. The *S*. *viridis* parent was taller than the *S*. *italica* parent in both trials, and 33% of the RILs in the greenhouse trial and 44% of the RILs in the field trial had heights that showed transgressive segregation by being either shorter than the *S*. *italica* parent or taller than the *S*. *viridis* parent (S1 and S4 Figs). In general, genotypes were taller in the greenhouse trial than those in the field trial.

Tiller number at the vegetative stage was measured in both trials but only one plant had a tiller in the plants destined for the field trial, presumably because vegetative measurements for this trial were taken three days earlier than for the greenhouse trial. Tiller number in the greenhouse trial ranged between zero and four tillers with the *S*. *viridis* parent having an average of 1.8 tillers and the *S*. *italica* parent having no tillers (S1 Fig). Eight RILs and the *S*. *italica* parent had no tillers in the greenhouse trial while 12% of the RILs had more tillers than the *S*. *viridis* parent. The phenotypic correlation between height and tillering in the greenhouse trial was significant and positive (S1 Fig).

#### Flowering stage

In both trials the *S*. *viridis* parent flowered in about half the time of the *S*. *italica* parent, and was around 25% the height of the *S*. *italica* parent or shorter, a reversal of their relative height compared to the vegetative stage. Changes in height rankings from the vegetative to the flowering stage were observed for many (but not all) RILs as well, with those RILs that were taller at the vegetative stage usually flowering earlier, and therefore being smaller at the flowering stage, than those RILs that took longer to flower. In addition, there was a significant positive correlation between the relative height of the RILs and the time it took them to flower in both trials (S2 and S5 Figs). Plants were taller at flowering in the greenhouse (33.5 ± 9.6 cm) than in the field (21.8 ± 6.5 cm). The RILs in the field trial took significantly longer to flower (34.5 ± 4.0 days) than those in the greenhouse trial (27 ± 3.7 days).

At flowering in both trials, every RIL had grown at least one tiller and about 50% or more of the RILs also had at least one aerial branch. In both field and greenhouse, the *S*. *italica* parent produced on average one tiller by the time the inflorescence on the main culm had emerged. In contrast, the *S*. *viridis* parent had an average of two tillers in the greenhouse and seven in the field. Significant differences between greenhouse and field are also reflected in the RIL population, where the average tiller number across all RILs in the greenhouse was 4.2 ± 2.2 and in the field 5.2 ± 2.2 tillers. Some genotype X environment interactions were observed for tiller number at flowering, with several genotypes being highly tillered in the greenhouse but with considerably less tillers in the field, and vice versa.

Aerial branches were only observed on the *S*. *viridis* parent and not on the *S*. *italica* parent, and average aerial branch numbers across all RILs at flowering were relatively similar for both trials (field 0.74 ± 0.5, greenhouse 0.76 ± 0.6 aerial branches). An interaction between environment (trial) and RIL for aerial branch number was observed, as with tillers. This interaction is more evident when tillers and aerial branches are combined as total branches, as some highly branched genotypes in one trial are much less branched in the other trial. Average total branches at flowering was slightly higher in the field (6 ± 2.4) than in the greenhouse (5 ± 2.4) (S2 and S5 Figs). The proportion of total branches that are tillers, expressed as a percentage, was similar between trials at flowering (greenhouse = 84.0 ± 12.8%, field = 86.7 ± 8.2%).

Height and tillering were significantly negatively correlated at flowering in both trials, whereas there was no significant correlation between height and number of aerial branches (S2 and S5 Figs). Height was significantly positively correlated with days to flowering in both trials. Days to flowering in the field was significantly negatively correlated with both tiller number and aerial branch number, whereas in the greenhouse days to flowering was significantly positively correlated with tiller number and showed no relationship with aerial branch number. There was a significant positive correlation between tiller and aerial branch number in both trials (S2 and S5 Figs).

The PCA analyses at flowering found two eigenvectors in each of the trials, one of which showed a negative loading for tillering and a positive loading for height and node number, and the other showing positive loadings for tillering and aerial branching, suggesting that a component of branching at flowering is independent of height (Table 1).

**Table 1:** PCA analysis of the relationship between architectural traits at both flowering and at harvest in each trial.

Stage	Variables	Greenhouse Components		Field Components	
		GH1	GH2	F1	F2
Flowering	Tillering	– 0.33	+ 0.75	– 0.58	+0.55
	Aerial branching		+ 0.83		+0.95
	Nodes	+ 0.92		+ 0.91	
	Culm height	+ 0.94		+ 0.91	
Eigenvalue (% variation explained)		1.92 (48.1%)	1.22 (30.5%)	2.26 (56.6%)	0.96 (24.1%)
Harvest	Tillering	– 0.41	+0.79		+0.90
	Aerial branching		+0.92		+0.76
	Culm height	+ 0.84	–0.38	+0.82	–0.45
	Tiller height (tallest)	+ 0.86		+0.84	
	Nodes	+0.59	–0.54	+0.56	–0.64
	Biomass	+0.73		+0.92	
Eigenvalue (% variation explained)		3.61 (60.2%)	0.89 (14.8%)	2.95 (49.1%)	1.66 (27.7%)

To simplify reporting of the results, trait loadings less than 0.3 are not shown. All analyses used transformed values for the traits, as detailed in the Materials and Methods.

#### Harvest stage

Plants in the field were harvested when 50% of the seeds on the main inflorescence were mature, and took an average of 70 ± 4.3 days to this point (range 62 to 88 days). Plants in the greenhouse were harvested when most of the seeds on all of the inflorescences were mature, taking an average of 103 ± 8.5 days to this point (range 78 to 121 days) (S3 and S6 Figs). In both trials we harvested plants in order of their maturation, to ensure that plants did not disintegrate before phenotypic measurements could be made, and to limit the impact of external factors such as herbivory (particularly in the field trial) or mechanical damage. In both trials *S*. *viridis* was harvested much earlier than the domesticated *S*. *italica*.

**Fig 2:**
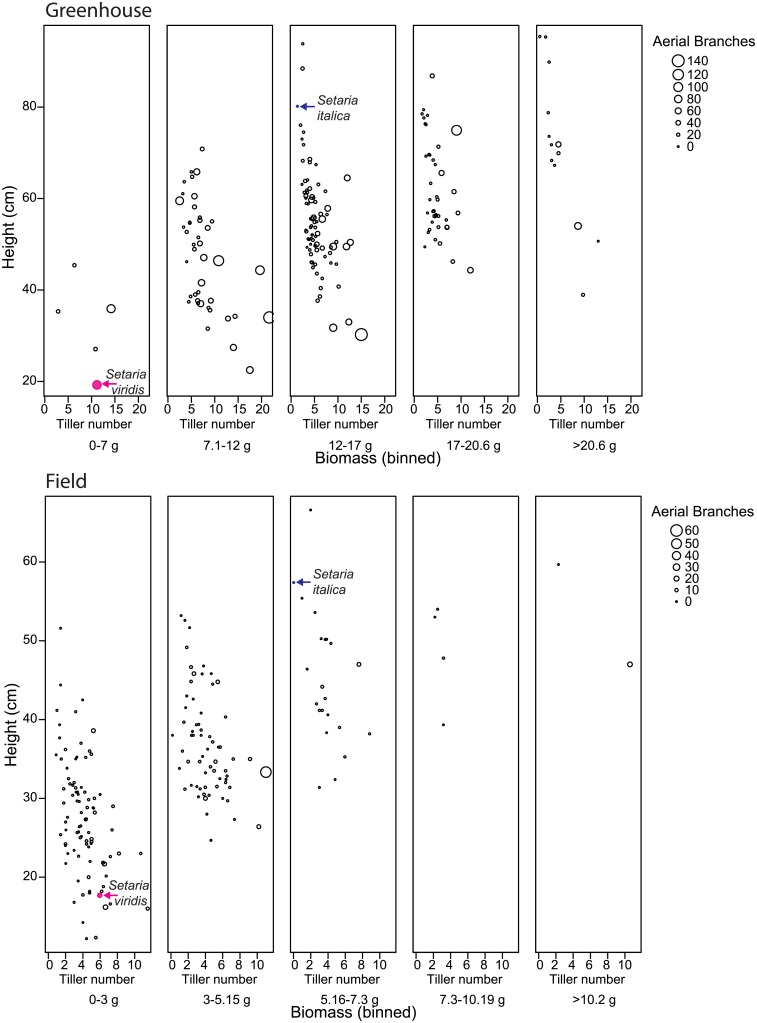
Relationships between biomass and plant architectural traits. Each panel represents a range of biomass values, with that on the left representing the lowest biomass and that on the right the highest. Height versus tiller number at harvest for each RIL is plotted in the appropriate panel depending on the biomass of the RIL. The size of the symbol is proportional to the number of aerial branches for the RIL (see legend at side of each figure). The values for *S*. *italica* and *S*. *viridis* are denoted by labeled arrows. A. Greenhouse, B. Field.

The average height of the *S*. *viridis* parent was about 25% the height of the *S*. *italica* parent, and average heights of both parents were taller in the greenhouse than in the field (Fig 2). This was also true for the RIL population, with an average plant height in the greenhouse of 55.4 ± 13.7 cm versus 33.7 ± 9.9 cm in the field. The height of the tallest tiller followed the same trend as for the main culm, being taller in the greenhouse (55 ± 13.7 cm) than in the field (23 ± 9.3 cm) (S3 and S6 Figs). The average number of nodes on the aerial portion of the culm in the RIL population was greater in the greenhouse (6.7 ± 1.2 nodes) than in the field (5.5 ± 1.1 nodes), and both parental genotypes had approximately one more culm node in the greenhouse than in the field. Overall, there were many shifts in plant height rankings observed across developmental stages for both parents and for most of the RILs in both trials (S7 Fig). Some of this is due to differences in flowering time, with earlier flowering plants being in general shorter than those that flower later (S3 and S6 Figs). However, this does not explain all of the differences, given that there is a wide range in height at any given flowering or harvest time, suggesting that differences in growth rate are also involved (S3 and S6 Figs). Thus, while there were a few RILs that consistently ranked amongst the shortest, medium or tallest across all three developmental stages, there were also RILs that shifted relative ranking in either direction over their development (S7 Fig).

In addition to being taller, plants were more branched at harvest/maturity in the greenhouse trial than in the field trial, primarily due to an increase in aerial branches. This contrasted with branching at flowering where more branches were observed in the field than in the greenhouse trial. The *S*. *viridis* parent had an average of 11.2 ± 2.2 tillers in the greenhouse and only 6.6 ± 3.4 in the field, while the *S*. *italica* parent had an average of 1.3 ± 0.9 tiller in the greenhouse and 0 in the field. The *S*. *viridis* parent had an average of 81.3 ± 22.5 aerial branches in the greenhouse and only 17 ± 7.6 in the field, while the *S*. *italica* parent never had aerial branches (Fig 1). Larger tiller numbers were observed in the greenhouse for the RIL population (6 ± 3.5 tillers) as compared to the field (4 ± 2.1 tillers) (S3 and S6 Figs). Average aerial branch number for the RIL population was quite different across trials, in the greenhouse it was 25 ± 31.3 versus only 3.4 ± 5 in the field. Some transgressive segregants produced more than 100 aerial branches in the greenhouse trial, with one of them producing almost 300 aerial branches. Differences in total branch numbers for the RILs followed the same trends as for tillers and aerial branches, with 31 ± 33.8 total branches in the greenhouse and 7.4 ± 6.3 total branches in the field (S3 and S6 Figs). The proportion of total branches that are tillers, expressed as a percentage, differed significantly between trials (greenhouse = 27.3 ± 14.4%, field = 63.9 ± 21.1%).

The relationships between the different traits at harvest followed the same general pattern as at flowering, with tillering and aerial branching positively correlated with each other, and these negatively correlated with height-related traits such as height of main culm, height of tallest tiller, and number of nodes, which are all positively correlated with each other (S3 and S6 Figs). Total biomass is negatively correlated with branching traits in the greenhouse trial and not significantly correlated with any branching trait in the field trial. Total biomass is positively correlated with height-related values in both trials (S3 and S6 Figs).

The PCA analyses at harvest found two components in both trials, with component one showing positive loadings on culm height, tallest tiller height, node number and biomass. In the greenhouse trial there was also a negative loading for tiller number. Component two showed positive loadings for tillering and aerial branching and negative loadings for culm height and node number (Table 1). These results suggest that there is a component of plant size variation explained by the negative relationship between plant height and branching, as well as a second component of plant size that can be independent of branching.

Total biomass produced was larger in the greenhouse than in the field, with the *S*. *viridis* parent producing 400% more biomass and the *S*. *italica* parent producing 100% more biomass in the greenhouse than in the field. The RILs also followed this trend with an average in the greenhouse of 14.5 ± 4.2 g compared to 3.4 ± 1.9 g in the field. The increase in biomass was not simply due to a longer growth period as the average biomass growth rate (total biomass divided by days to harvest) for the RIL population was higher in the greenhouse trial than in the field, (0.14 ± 0.04 g/day vs. 0.05 ± 0.03 g/day, respectively), with the parental genotypes following the same trend. In both trials the *S*. *italica* parent produced more than twice the biomass and at more than twice the rate of the *S*. *viridis* parent (S3 and S6 Figs).

Correlations between biomass and traits at all stages of the lifecycle were calculated in order to identify those traits that are predictive of high biomass. Height at flowering and at harvest, together with height of the tallest tiller and node number were all good predictors of final biomass (S1 Table). Branching traits were negatively correlated with biomass in the greenhouse trial but not correlated in the field trial (S1 Table).

In order to visualize how biomass varied with height and branch number, RILs were first ranked and binned by biomass values; then tillers versus height were plotted for each bin. Aerial branch number was incorporated into this analysis by varying the size of the symbol in proportion to the number of aerial branches (Fig 2). Recombinant inbred lines with 60% more biomass than the *S*. *italica* parent were binned in the highest yielding group (biomass higher than 20.6 g in the greenhouse and biomass higher than 10.2 g for the field trial). The biomass binned plots have *S*. *viridis* in the lowest biomass bin and *S*. *italica* in the middle bin in both trials. Thirteen RILs produced 60% more biomass than that produced by the *S*. *italica* parent in the greenhouse trial and two did so in the field trial. The two highest biomass RILs identified in the field are both more than 40 cm tall, with one RIL having only 3 branches (all tillers) while the other has 29 branches (including 11 tillers). The greenhouse trial showed an even wider variation in plant architecture, with average total branch numbers for RILs in the highest biomass bin ranging between 5.5 and 78.2. The RIL with an average of 5.5 total branches included 3 tillers, and the RIL with an average of 78.2 total branches included 9 tillers (Fig 2).

### QTL analyses

**Fig 3:**
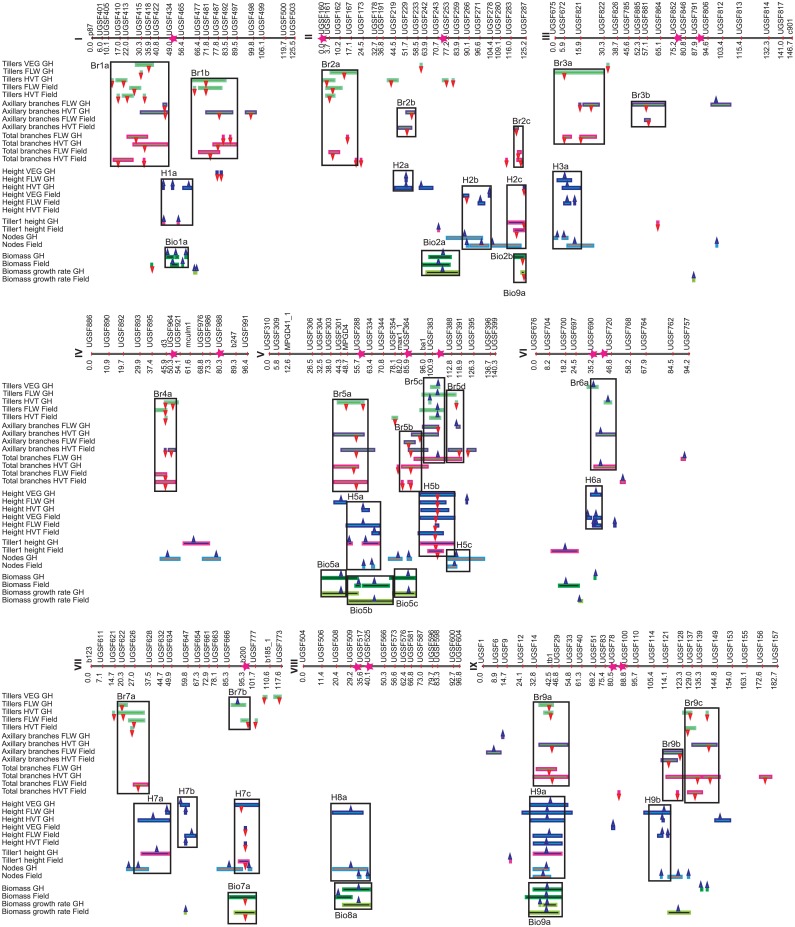
QTL map. QTL map showing the QTL locations for multiple traits on the nine chromosomes of *Setaria*. Boxes represent genomic regions where more than one branching or height or biomass QTL co-locate. Magenta stars on the line representing the chromosome represent the maximum LOD positions of flowering time QTL identified for the same mapping population by Mauro-Herrera et al. (2013). GH refers to the greenhouse trial, Field to the field trial.

QTL analysis was performed for 14 traits in the greenhouse trial and for 13 traits in the field trial (Fig 3, S2 Table). Named QTL regions designate genomic locations where QTL from more than one trial and/or developmental stage co-located.

#### Height

For the greenhouse trial there were 7 height QTL identified at vegetative, 10 at flowering, and 13 at harvest stages, explaining 63.9, 60.9 and 76.3% of the phenotypic variance, respectively. For the field trial there were 7 QTL identified at vegetative, 12 at flowering, and 5 at harvest stages, explaining 78.1, 69.4 and 44.4% of the phenotypic variance. *Setaria viridis* contributed the largest effect QTL (in QTL region H5b), which was identified at all three stages in both trials, and explained most of the variation at the vegetative stage (33–61%), and substantial amounts of variation at flowering (5–14%) and harvest (15–20%). *Setaria italica* contributed a second consistent QTL on chromosome IX (in QTL region H9a), found at the vegetative stage in the greenhouse trial and at flowering and harvest in both trials, and explaining between 7.6% and 15.5% of the phenotypic variance. Other QTL were identified across developmental stages but only in a single trial. The *S*. *viridis* alleles increased height at half of the QTL identified at the vegetative stage (including the one in QTL region H5b with the highest phenotypic effect), but only a single QTL at flowering, and two at harvest. QTL affecting flowering time identified from a previous study (Mauro-Herrera 2013) colocalized with many of the height QTL including the large effect QTL in H5b (Fig 3).

#### Tillering

For the greenhouse trial there were 7 tillering QTL identified at vegetative, 7 at flowering, and 19 at harvest stages, explaining 43, 57 and 89.6% of the phenotypic variance, respectively. In the field trial at the vegetative stage tillers were not yet present, while at flowering and harvest there were 9 QTL identified explaining 47.4 and 41.8% of the phenotypic variance, respectively. Several tiller QTL were identified that were shared across developmental stages and/or trials, including those in QTL regions Br1a, Br1b, Br2a, Br4a, Br5c, Br7a, Br9a and Br9c; with the one in Br5c being the most consistent and explaining some of the largest proportions of phenotypic variance (8.5% at the vegetative stage in the greenhouse, and between 4.5 and 23.6% at the other two stages). Alleles from the most-branched parent, *S*. *viridis*, increased branching for all QTL except for those in QTL regions Br5c, Br6a, and a single QTL identified on chromosome VII.

#### Aerial branching

Neither trial exhibited aerial branching at the vegetative stage. In the greenhouse trial there were 8 aerial branching QTL identified at flowering, and 9 at harvest, explaining 46.8 and 63% of the phenotypic variance, respectively. In the field trial there were 4 aerial branching QTL identified at flowering and 9 at harvest, explaining 29.7 and 42.4% of the phenotypic variance, respectively. Aerial branching QTL in QTL region Br3b were identified in both trials only at flowering, explaining 5.9–9.1% of the phenotypic variance, and QTL in QTL regions Br2b and Br5a were identified in both trials only at harvest, explaining an average 4.5% of the variation for QTL in QTL region Br2b, and an average 12.5% of the variation for QTL in QTL region Br5a. In most cases aerial branching QTL overlapped with tillering QTL, although exceptions occur for QTL regions Br2b, Br3b, Br5b, and Br9b. Overall, the *S*. *viridis* alleles increased aerial branch number at flowering at 7 of the 11 identified QTL, and increased aerial branching at harvest at all but one QTL (in Br6a) of the 15 identified aerial branch number QTL.

#### Total branches

QTL identified for total branch number in general overlap with QTL for either tiller number or aerial branch number, with the exception of a total branch number QTL identified at flowering in the greenhouse trial on chromosome VI, and five others identified at harvest (one of them in the greenhouse trial and the other four in the field trial) on chromosomes II, VI and IX.

#### Biomass

Twelve QTLs in the greenhouse trial and ten QTLs in the field trial were identified for biomass, explaining 57% and 64.2% of the phenotypic variance respectively. Three QTLs were identified in both trials (in QTL regions Bio1a, Bio2a and Bio9a), with effects explaining between 3% (in Bio1a) and 14% (in Bio9a) of the phenotypic variance, with the *S*. *italica* alleles increasing biomass. *Setaria viridis* alleles increased biomass for three QTLs, including a large effect QTL on chromosome VII in the field trial that explained 14% of the phenotypic variance (in QTL region Bio7a). For all the other biomass QTL *S*. *italica* increased biomass. For the most part, biomass QTL overlapped with QTL for culm height, height of the tallest tiller, and/or node number.

#### Biomass growth rate

Nine QTL for biomass growth rate were identified in the greenhouse trial and seven in the field trial, explaining 48.3 and 48.9% of the phenotypic variance, correspondingly. The biomass growth rate QTL in QTL region Bio7a had the highest phenotypic effect. It was detected in the field only and explained 14.1% of the variance, with the *S*. *viridis* alleles increasing biomass growth rate. The *S*. *viridis* alleles also increased biomass growth rate in the Bio2b QTL region, though the effect was small (3.8%) and it was identified in the greenhouse only. For all other QTL the *S*. *italica* alleles increased biomass growth rate, with a phenotypic effect between 3.1 and 10.6%.

#### Nodes

A total of fourteen QTL for nodes on the aerial portion of the culm were identified in each trial, explaining 68.3 and 68.6% of the phenotypic variance in the greenhouse and field trial, respectively. The *S*. *italica* alleles had a positive effect for every node QTL identified. Four QTL regions were identified in both trials (H2b, H5c, H8a, and H9a).

#### Height of the tallest tiller

In the greenhouse trial ten QTL were identified for the height of the tallest tiller, while in the field trial six QTL were identified, explaining 76.7 and 47.9% of the phenotypic variance. The *S*. *viridis* alleles increased the height of the tallest tiller at the two QTL regions identified in both trials (H2c and H5b). QTL in QTL region H5b had the highest phenotypic effect in both trials, explaining 10.6 to 24.7% of the phenotypic variance, and are in the same QTL region (H5b) that has the QTL with the greatest and most consistent effect on culm height. The *S*. *viridis* alleles also increased height at two other QTL identified in a single trial, on chromosomes III (a single QTL) and VII (in QTL region H7c). The *S*. *italica* alleles increased the height of the tallest tiller for the ten other QTL.

#### Parental contribution to plant architectural traits

Both parents have an equal number of alleles increasing height at the vegetative stage although the phenotypic variance explained by the *S*. *viridis* alleles is much higher than that explained by the *S*. *italica* alleles (46.8% as compared to 17.1% in the greenhouse and 66.4% as compared to 11.7% in the field). At flowering, *S*. *viridis* alleles increase height at H5b (which also explained the most variation at the vegetative stage), with *S*. *italica* alleles increasing height at all other QTL. At harvest *S*. *viridis* alleles increased height at three QTL (in QTL regions H2c, H5b and H7c), including one that explained the most phenotypic variance of any of the QTL identified (in QTL region H5b). For all other QTL the *S*. *italica* alleles increased height. Both *S*. *viridis* and *S*. *italica* parents contribute alleles to the height of the tallest tiller at harvest, but the *S*. *italica* parent contributed all of the alleles increasing node number on the main culm.

Overall, the *S*. *viridis* parent contributed most of the alleles increasing tiller and aerial branch number, and contributed the alleles with the strongest effects for aerial branching at flowering (QTL in QTL regions Br3b, Br5b and Br5c), and at harvest (QTL in Br5a and Br9a). *Setaria italica* had a strong effect QTL for aerial branching at flowering on chromosome IX (not in a named QTL region), and at harvest (QTL in Br6a). *Setaria italica* also alleles increased tiller number in QTL region Br5c, a QTL that was identified at the three developmental stages and in both trials, and at Br6a, which includes QTL for branching at vegetative and harvest stages. *Setaria viridis* alleles also have an effect on branching in QTL region Br5c but appear to be influencing aerial branching rather than tillering. There were seven other QTL for which the *S*. *italica* alleles increased branching but they were identified only in a single trial and developmental stage.

Biomass is primarily increased by *S*. *italica* alleles although *S*. *viridis* alleles increase biomass for three QTL which explained between 4.1 to 14% of the phenotypic variance for this trait (S2 Table).

#### Epistasis

A single significant epistatic interaction was detected at the Bonferroni adjustment to the experiment-wide error rate of P < 0.05 (with a P-value of P < 2.3 x 10^−7^). This significant interaction for aerial branches at harvest was detected in the greenhouse trial, between markers UGSF886 and UGSF303 (P = 1.48E-07) and explained 13.3% of the phenotypic variance. UGSF886 is on top of chromosome IV, in a region devoid of any QTLs, and UGSF303 is on chromosome V, and falls within the Bio5a region. Another almost significant epistatic interaction (P = 2.42E-07) was also identified between these same markers for total branches at harvest. No significant interactions were detected in the field trial.

#### Comparison of QTL results with other studies

Several overlapping QTL were identified between this study and studies by Doust et al. (2004), Doust et al. (2006), and Mauro-Herrera et al. (2013) which also used this cross [21, 22, 79]. There were multiple shared tiller number QTL, with QTL in QTL regions Br1b, Br3a, Br5a, Br5c and Br9a being identified in one or both of the previous studies. There were also many corresponding QTL for aerial branch number, such as the one in QTL region Br1a, which was found at flowering and harvest in the greenhouse trial and in Doust et al. (2006). Another aerial branch number QTL in Br3b was also identified in Doust et al. (2004). Several of the QTL for aerial branch number on chromosome V identified in the present study were identified in one or both of the previous studies. As expected, there are also a number of QTL that were not shared between the studies. It is possible that more QTL may be shared between this study and the two previous QTL studies [21, 22] but the lack of sequence information for many of the RFLP markers used in the earlier studies precludes accurate alignment of the RFLP-based maps with the present SNP and SSR map. Comparisons of the QTL positions with those of flowering time QTL identified in Setaria [60], using the same F_7_ RILs and genetic map, indicate that flowering time loci co-locate with six QTL for height and seven for branching, with four of these QTL affecting both height and branching.

Comparison of QTL identified in this study with genome-wide association studies in foxtail millet [79] found several GWAS loci within QTL regions for branch number, tiller number and node number, but no GWAS loci within height QTL regions. Two regions identified as having undergone selective sweeps in the Jia et al. study [79], one on chromosome II for tiller number and one on chromosome VII for node number, fell within QTL for those traits found in the present analysis.

QTL identified in this study for height, biomass, and branching were also compared with several published studies in sorghum, maize, and switchgrass where genome comparisons of QTL locations could be assessed. Some of the height QTL detected in Setaria colocalize with height QTL found in maize, including the largest effect QTL identified in a recent study using the Nested Association Mapping panel [80] (maps to an unlabeled node and tiller height QTL on Setaria chromosome IV). Setaria height QTL regions, including H1a, H2a, H3a, H5b and H5c, also co-locate with about half of the QTL identified in various sorghum QTL studies [52]. A similar situation is found for comparisons between Setaria and switchgrass QTL studies. Daverdin et al. (2015) [81] identified QTL in a cross between upland and lowland ecotypes of switchgrass, and identified nine height QTL on the female map and eleven on the male, of which nine colocalized with height QTL regions identified in Setaria (H1a, H2b, H2c, H5a, H5b, H7a, H7b, H9a and H9b). Daverdin et al. (2015) also identified nine QTL for biomass in the female and eight QTL for biomass in the male map, of which five colocalized with Setaria biomass QTL. These five biomass QTL also colocalized with five height QTL that were found in both the switchgrass and Setaria studies, reinforcing the strong role that height plays in biomass production. Another switchgrass study by Lowry et al. (2015) identified five height QTL in a cross between two lowland ecotypes, of which two colocalized with height QTL identified in Setaria [48]. Lowry et al. (2015) identified a single major QTL for biomass that was also identified in both the Setaria analysis and Daverdin et al. (2015) [48, 81]. In Setaria, this QTL region (Bio9a) accounts for 6% of the phenotypic variation in biomass in the greenhouse and 14% in the field. Many of the height QTL identified in that and other studies do not colocalize with Setaria height QTL, but this is expected given that our population likely segregates for only some of the many existing QTL in Setaria.

Tillering QTL regions that colocalize with those in sorghum include Br1b, Br3a, Br7a, Br7b, and Br9b [17]. Aerial branching QTL in sorghum [17] colocalize with Setaria QTL regions Br5a, Br9b, and Br9c. Only two of the ten Sorghum QTL [17] do not colocalize with Setaria branching QTL regions. In the one switchgrass QTL study that addressed the genetic control of branching, no QTL could be identified, suggesting strong environmental effects or many genes with small effects on branching in this species [48].

#### Candidate genes

QTL regions were scanned for relevant candidate genes as described in the Materials and Methods (S3 Table), and candidate genes were identified in both height and branching QTL regions. In region H5b, for which the *S*. *viridis* allele increased height, a GIBBERELLIN 20-OXIDASE like gene (GA 20-ox-like)[82] was identified, that is the ortholog of the rice semi-dwarfing gene *SD-1* [83, 84], and which lies within 1000 bases of the maximum LOD peak of a number of the height QTL in this QTL region. Analysis of the coding region of the *S*. *italica* and *S*. *viridis* orthologs (Si001573m and Sevir.5G410400 respectively) in Phytozome [66, 85] suggests that both copies are functional, but that there are two amino acid changes in *S*. *italica*, one an aspartate to a glutamate in the first exon, and the other an aspartate to an alanine in the third exon. This QTL region also co-locates with a QTL region for branching (Br5c, for which *S*. *italica* increased tiller number) that includes the candidate genes *Lnt1*, which affects axillary meristem initiation and/or axillary bud development [86] and *OsMADS57*, which interacts with *OsTB1* to control axillary bud outgrowth [87].

A second genomic region where height and branching QTL regions co-locate is that of H9a and Br9a, with the *dwarf8* gene [88] being a candidate for height and *knotted1* (involved in meristem maintenance and branching modification) [89], *phytochrome B* (which represses *tb1* to promote axillary bud outgrowth) [90], and *tb1* (which represses tiller/axillary bud outgrowth) [91] (S3 Table) being candidates for branching. A third QTL region is on chromosome V, where QTL regions H5a and Br5a co-locate. The QTL in these regions are not consistently expressed across developmental stages or trials, and max LOD peaks for branching and height are in very close proximity but do not exactly overlap. A candidate gene for the Br5a QTL region is *corngrass1*, which in maize encodes two tandem miR156 genes, and where the mutant produces tillers in the axil of each leaf [92], while for H5a a candidate gene is SHOOTLESS2, a gene involved in initiation and maintenance of the shoot apical meristem [93]. Other height QTL regions contained genes from the gibberellin pathway, including GIBBERELLIN 2-OXIDASE (GA2ox7) [94] (region H7b), and GIBBERELLIN 2-OXIDASE (GA2ox1) [94] (region H7c).

Some branching QTL regions are in locations that are devoid of height QTL (e.g. regions Br1a, Br1b, Br2a, Br3b, Br4a and Br9c). Candidate genes in these QTL regions include *LATERAL SUPPRESSOR* (*LAS*)/*MONOCULM1-like* (*MOC1-like*), *GIBBERELLIN 2-OXIDASE* genes, *REDUCED SHOOT BRANCHING 1 (RSB1)*, *OsKN3/KNOTTED1-like*, *OsPIN3b*, *DWARF3 (DWF3)/MAX2 HOMOLOG (D3)*, *REDUCED CULM NUMBER1 (RCN1)*, *SHADE AVOIDANCE 1 (ATDWF4)/ DWARF4 (D4)*, *DWARF88 (D88)/DWARF14 (D14)*, and *grassy tillers1 (gt1)*. These genes are involved in different steps of meristem initiation and bud elongation.

## Discussion

### Changes in plant architecture across developmental stages and their effect on final biomass

*Setaria viridis* and *S*. *italica* differed in their height and branching patterns at all developmental stages. *Setaria viridis* was significantly taller than *S*. *italica* at the single time point measured during the vegetative stage (14 days after sowing in the greenhouse trial and 11 days after sowing in the field trial), and, in the greenhouse trial, had already started to produce tillers. This single vegetative time-point represents quite different stages in the lifecycle of the two parents: *S*. *viridis* was approximately 42–70% of the way towards flowering (as it took 20–26.5 days to flower), whereas *S*. *italica* was only 20–35% of the way towards flowering (as it took 40–54 days to flower). This strongly suggests that the life-history strategy of the wild parent is to grow and mature quickly. At flowering, the relative heights of the parents changed, with *S*. *italica* being at least three times as tall as *S*. *viridis*, but taking twice as long to get to that stage. Despite the greater time to flowering and increase in height, *S*. *italica* had fewer tillers than *S*. *viridis*, but these were each much thicker than those of *S*. *viridis*. In addition, many *S*. *viridis* plants had started producing aerial branches by flowering, especially in the field trial, whereas aerial branches were never seen in *S*. *italica*. At harvest, *S*. *italica* was still much taller, much less branched, and much heavier than *S*. *viridis* (S1 Fig).

The growth patterns described for the parents were also seen in the RILs, with several showing a pattern of accelerated growth and quicker maturation, so that their size rankings switched from being amongst the tallest in the vegetative stage to being amongst the smallest in the flowering and harvest stages. Others showed an opposite pattern, being amongst the smallest (i.e. slowest growing) at the vegetative stage but being amongst the tallest genotypes at flowering and harvest. However, some RILs grew relatively quickly throughout developmental stages, and still attained a large size whilst others grew more slowly and remained small, suggesting that several loci controlling these growth responses are segregating within the RIL population. The PCA analyses at flowering show a negative correlation between tillering and height in the first component for both trials, and a positive relationship between tillering and aerial branching in the second component. Thus both trials show a strong negative correlation between plant size (height) and branching as well as significant variation in tiller and aerial branch number independent of size. This is supported by the QTL analysis which show at least four regions where branching and height QTL regions overlap, as well as multiple QTL regions where branching and height QTL regions appear independently.

The PCA analyses at harvest show similar patterns of correlations as those at flowering. The first component in both trials shows strong positive loadings for culm height, tiller height, node number and biomass while the second component shows a negative relationship between tiller and aerial branch number on the one hand and culm height, tallest tiller height, and node number on the other. Within each, branching and height trait groups, there were strong positive bivariate correlations between individual traits. The greenhouse trial also has a negative loading for tiller number in the first component, which may be because there was a late burst of tillering in many RILs in that trial, producing masses of fine short tillers in those genotypes.

There were also plant architectural differences due to the different environmental conditions of the two trials. Both parental genotypes were shorter in the field than in the greenhouse, and both parents had more branches and biomass by harvest time in the greenhouse than in the field. This was partly because the greenhouse trial was left to grow for a longer period of time after flowering than the field trial, but was also due to faster biomass growth rates for both parents in the greenhouse compared to the field (S1 Fig). The increased growth rate in the greenhouse may be due in part to the continual application of dilute fertilizer in the irrigation, as opposed to the one-time application of fertilizer employed in the field trial.

Biomass may also be affected by stem thickness, as we observed that tillers of *S*. *italica* are thicker than those of *S*. *viridis*, and that stem thickness both segregates within the mapping population, and varies between different orders of branching. Furthermore, our qualitative observations suggest that, in general, tillers that are present at flowering are markedly thicker than those produced later, and were significantly correlated with final biomass in the greenhouse trial. Biomass is not correlated or is negatively correlated with branching at harvest, possibly because many of the branches produced late in development (mostly aerial branches) are thin and short, and contribute little to the biomass. Biomass is more highly correlated with height than with branching, as shown by the bivariate correlation coefficients and the PCA results, although branching can play a part in producing high biomass in some RILs. RILs in the highest biomass category varied from having three tillers and no branches to nine tillers and 69 branches, indicating that a range of branching patterns can produce comparably high biomass plants at maturity, as seen in Fig 2.

### QTL results

The QTL analysis revealed a number of regions where biomass QTL co-located with QTL regions controlling height and/or branching. Co-located biomass and height QTL regions included Bio1a/H1a, and Bio8a/H8a, while biomass QTL regions that co-locate with both height and branching QTL regions include Bio2b/H2c/Br2c, Bio5b/H5a/Br5a, Bio7a/H7c/Br7b and Bio9a/H9a/Br9a. This suggests that these regions either contain multiple linked QTL/genes controlling these traits or that there exist pleiotropic loci that mediate both branching and height at the same time. Examples of such pleiotropic loci include, for example, the rice branching mutants *HTD1* and *D3* in the strigolactone pathway, which have shortened stature and increased branching compared to wild type [34–39]. Only one biomass and branching QTL region combination, Bio5c/Br5b, does not co-locate with a corresponding height QTL. This biomass QTL is only found in the greenhouse trial, where it co-locates with QTL for node number, and where the *S*. *italica* allele is associated with increased biomass as well as nodes, and a reduction in branching. The increase in biomass in this case may be due to an increase in leaf number associated with the increase in nodes.

QTLs identified in the present study for traits measured at multiple stages (specifically aerial branches, tillers and height) can be divided into those that are common between trials and appeared in more than one developmental stage, those that are common between trials but are specific to a developmental stage, those that are confined to one trial but found at more than one stage, and those that are both trial and stage specific (these last two likely contributing to environmental specificity/phenotypic plasticity). There are many QTL in the last category with nine (18%) out of the 50 branching QTL identified in the greenhouse trial uniquely identified at a specific developmental stage (three at vegetative, one at flowering and five at harvest), and four (13%) branching QTL of the 31 identified in the field trial being unique to this trial and at flowering (two) or harvest (two). For height, 10 (33%) height QTL were uniquely identified in the greenhouse at one of the developmental stages; whereas two (8%) height QTL were uniquely identified in the field at a single developmental stage. Therefore, a moderate number of plant architectural QTL are both environmentally and developmental stage specific, ranging between 13–18% for branching and 8–33% for height.

There were four branching QTL and two height QTL that were specific to a single trial/environment but found at multiple developmental stages, of which all but one were in the greenhouse trial. These included aerial branching QTL in QTL regions Br1a, Br5c, Br9b, and tiller QTL in Br9a. The height QTL are in QTL regions H2a and H7a. These QTL reflect environmental differences between the two trials and possibly are involved in the plastic response of plant architecture.

Those QTL that were found in both trials but at only one developmental stage were all branching QTL and were located in QTL regions Br2b, Br3b and Br5a (affecting aerial branch number at flowering or harvest) and in Br9c (affecting tillers at harvest). The timing of these QTLs’ appearance accords with our field observations and with observations in the literature [21] that suggest that aerial buds elongate to form branches primarily after flowering, so that variation in branching becomes increasingly pronounced as the plants develop towards harvest. Many QTL are found across multiple stages in both trials, with seven height QTL in QTL regions H3a, H5a, H5b, H6a, H7b, H9a and H9b and five tiller QTL, in QTL regions Br1a, Br2a, Br4a, Br5c and Br7a falling in this category. There are no QTL that are specific for just the vegetative stage, even though there are a number of QTL specific for either flowering and/or harvest stages. In part this is because the branching traits (specifically tillers) had only just started to develop at the vegetative stage, and were only measured in one trial. QTLs for height at the vegetative stage are included in QTL regions that also contained QTL identified at flowering and/or harvest stages, emphasizing the relative stability of the height QTL. These results are similar to those reported in rice, wheat, maize, and switchgrass where the analysis of tillering and plant height QTL across different developmental stages also led to the identification of stage specific QTLs, as well as QTLs with different effects at different developmental stages [48, 61, 62, 75, 95].

There is a strong relationship between height and branching QTL, especially for those QTL with the greatest effects. The two height QTL regions with strong phenotypic effect QTLs that were consistently identified across developmental stages, H5b (for which *S*. *viridis* increased height) and H9a (for which *S*. *italica* increased height), both co-locate with strong effect branching QTL regions (Br5c, Br9a). The cause of the increase in height at each QTL region may well be different as a node number QTL co-locates with the height QTL in QTL region H9a, suggesting that the increase in height contributed by *S*. *italica* allele at this locus may be due to an increase in number of nodes, while there is no co-located QTL for node number near region H5b, suggesting that the *S*. *viridis* allele is affecting internode length rather than the number of internodes. A strong candidate gene for H5b is a GIBBERELLIN 20-OXIDASE like gene (GA 20-ox-like) [82], which lies within 1000 bp of the max LODs of many of the height associated QTL within H5b. This gene is the ortholog of the *SD-1* locus in rice, whose semi-dwarfing phenotype was the basis of breeding efforts for the green revolution [83, 84]. The action of this gene in rice is to affect internode length, which is consistent with the inference that the *S*. *viridis* allele affects internode elongation, rather than node number. This QTL region also co-locates with a QTL region for branching, where the Setaria allele reduces branching. This is consistent with an increase in gibberellin upregulating *tb1* expression and thus suppressing branching, as also reported in rice [44]. No biomass QTL region co-locates with QTL regions H5b/Br5c, suggesting either that biomass is not strongly affected by internode elongation or that the strong opposite phenotypic effects on branching and height may have counteracted each other, resulting in similar effects on biomass from different allele combinations.

The QTL region H9a contains height QTL identified in several stages in both trials while the co-located QTL in Br9a contains tiller and aerial branch QTL identified only in the greenhouse trial. In this region a consistent biomass QTL in region Bio9a co-located with H9a and Br9a, with *S*. *italica* increasing biomass. Node number QTL co-locate with the height QTL in the QTL regions, suggesting that the increase in biomass may be due to an increase in node and associated leaf number. In each of these strong height/branching co-located QTL regions one parent provides the allele that increases height and node number whilst the other contributes the allele that increases branch number. This is in agreement with the negative phenotypic correlation found between height and branching.

A third QTL region with an opposite effect between height and branching is on chromosome V, where H5a and Br5a co-locate. Here the *S*. *italica* alleles increased height while the *S*. *viridis* alleles increased branching of both aerial branches and tillers. The QTL in these regions are not consistently expressed across developmental stages or trials, and max LOD peaks for branching and height are in very close proximity but do not exactly overlap. The biomass QTL region that most overlaps with these QTL is Bio5b where the *S*. *italica* alleles increase biomass. Lastly, some branching QTL regions are in locations that are devoid of height or biomass QTL (e.g. Br1a, Br1b, Br2a, Br3b, Br4a and Br9c).

Six of the height QTL and seven of the branching QTL co-locate with QTL reported for flowering time [60] (Fig 3), suggesting that there is an interaction between flowering time and plant architectural traits. This is not surprising as architectural traits develop over the lifetime of the plant, and the transition to flowering limits leaf production and branching from that branch.

#### Comparisons of QTL results with previous studies

Previous studies in Setaria using essentially the same population (F_3_ in those studies, F_7_ in this study) gave relatively similar results, although the size of QTL intervals in the present analysis are smaller than in the earlier studies [21, 22]. Two regions in this study, for tillering and number of nodes on the culm, corresponded to regions of low diversity in a GWAS study of Chinese accessions of *S*. *italica* (foxtail millet), suggesting that the QTL may be in regions where there has been a selective sweep [79]. These two regions may indicate regions selected upon during initial domestication rather than later improvement, as they are maintained in all *S*. *italica* lines. In combination, the QTL and GWAS results are intriguing, as they suggest that the greater yield associated with domestication is primarily the result of stacking more nodes plus leaves on the main culm and by restricting the growth of side branches. The additional nodes and leaves also account for the increase in biomass, although differences in thickening of the culm and tillers may also be a factor. However, it is not clear that vegetative plant architecture itself was necessarily under direct selection during domestication, as changes in architecture may be the result of selection for larger inflorescences and/or larger seed.

Comparisons of QTL results with other cereal grasses indicate that there are syntenous genomic regions that share QTL for several of the traits studied. The synteny showed by these regions may indicate that both the genetic networks and their phenotypic effects have been conserved throughout grass evolution. One of these that is recognized as important in the evolution of maize is the region containing the *tb1* locus [29, 95, 96], which appears to be a central transducer of multiple signal pathways, and acts to repress bud elongation when upregulated [29, 97]. In both maize and in this Setaria mapping population, the region containing the *tb1* interval is identified as a QTL region (Br9a in this study), although it explains relatively little variation in branching in Setaria studies to date, and in this study is only significant in the greenhouse trial. The reverse is true in maize, where in many crosses it is a major domestication locus. These results highlight the significance of conducting branching studies in different systems to fully explore the effect of different genetic pathways involved in the development of plant architecture. Interestingly, a second QTL region in Setaria, orthologous to that identified as being epistatic to the *tb1* locus in maize [97], appears to be more important in explaining the variation in tiller number in Setaria (Br5b). The region that contains the *tb1* locus also has a strong effect on height (H9a), possibly due to a linked locus such as *dwarf8* [98–100]. More effort will be required to dissect the effects of this region on height and branching in Setaria.

## Conclusions

In this study, we have dissected the relationship between height and branching in Setaria across three developmental stages, using an F_7_ RIL mapping population derived from a cross between domesticated *S*. *italica* and wild *S*. *viridis*. In general, our results show that height related traits are negatively correlated with branching traits, though we also identified branching QTL regions that did not co-locate with height QTL regions. A major component controlling height in several QTL regions appears to be the number of nodes on the main culm, with those plants having more nodes being taller and taking longer to reach flowering. For instance, one of the two major QTL for height co-locates with QTL for node number, suggesting that the control of height can be by changing the number of nodes. In this QTL, *S*. *italica*, which has more nodes, has the alleles increasing height. The other major height QTL accounts for a large amount of the variation in height at the vegetative stage, where *S*. *viridis* is taller than *S*. *italica*, and where the alleles increasing height come from *S*. *viridis*. It does not co-locate with node number QTL, making it likely that this QTL controls internode length rather than node number, a possibility that is further reinforced by it also having a strong effect at harvest, when height differences can only be accomplished by elongation and not by addition of more vegetative nodes. In this region is a candidate gibberellin oxidase that is the ortholog of the rice *SD-1* semi-dwarfing locus, which controls internode elongation. In these two QTL regions the negative relationship between height and branching is apparent as alleles of one parental genotype increased height while the alleles of the other parental genotype increased branching.

The PCA analyses at flowering revealed that one component consists of a negative relationship between height and branching, whilst the other component consists of variation in branching. At harvest, the negative relationship between height and branching is conserved but the first component consists of variation only in height traits (in the field) and height traits plus tillering (in the greenhouse). In both trials height traits such as culm height, tiller height, and number of stem nodes are highly correlated with biomass. Bivariate correlations between biomass and branching are not always significant and usually negative, although there are many ways in which height and branching can be combined to allow high accumulation of biomass. In all cases tillers contribute more to biomass than aerial branches.

QTL analyses support a close relationship between height and branching, with a number of QTL regions simultaneously increasing branching and decreasing height (or vice versa). However, some branching QTL regions do not co-locate with height QTL regions (or vice versa). Therefore the genetic control of plant architecture could be made up of both specific and pleiotropic loci. Distinguishing pleiotropic loci from multiple linked loci within a QTL region will require much larger mapping populations, and fine-mapping of individual QTL regions. Furthermore, some co-locating branching and height QTL regions are not associated with QTLs for biomass, suggesting that the differences in height and branching may be mediated in such a way as to conserve overall biomass.

There were a few trait QTL that were found in both trials at a specific stage. Most of these QTL were for aerial branches, indicating that aerial branching is under developmental control. Many tiller QTL and most height QTL were found at multiple stages, whether or not they were found in both trials. This indicates a conserved genetic network controlling plant architecture throughout development, as well as environmental variation that influences QTL detection. Developmental and environmental variation in both branching and height may also be in response to changes in intra-plant competition and shading as the plants grow.

Comparative mapping of Setaria QTL against those identified from studies in maize, sorghum, and switchgrass, showed several QTL regions that overlapped. This could be evidence that there is a conserved genetic pathway controlling plant architecture in panicoid grasses. Some of these are likely conserved across grasses as a whole, based on the presence of orthologs of branching and height genes functionally characterized in rice. The candidate genes identified in Setaria QTL will be the basis for further exploration of the genetic basis of plant architectural development in Setaria. Additional experiments that further explore differences in growing conditions will also help to dissect how environmental factors affect architectural changes over the lifetime of the plant. The results presented here should be useful for breeding desired plant architectures, such as genotypes that can grow at various densities but still efficiently produce both grain and biomass.

## Supporting Information

S1 Fig Histogram distributions and correlations between traits at the vegetative stage in the greenhouse. Histograms are shown for each trait, using untransformed data. Scatter plots with Spearman’s rho correlations are shown for all pairs of traits. The position of values for the parents of the RIL population are shown in each histogram and scatter plot, with a magenta star for *S*. *viridis* and a blue star for *S*. *italica*. P-values are displayed for correlations that are significant, * P < 0.05, ** P < 0.01, *** P < 0.001.
(EPS)

S2 Fig Histogram distributions and correlations between traits at the flowering stage in the greenhouse. Details as for S1 Fig.
(EPS)

S3 Fig Histogram distributions and correlations between traits at the harvest stage in the greenhouse. Details as for S1 Fig.
(EPS)

S4 Fig Histogram distributions and correlations between traits at the vegetative stage in the field. Details as for S1 Fig.
(EPS)

S5 Fig Histogram distributions and correlations between traits at the flowering stage in the field. Details as for S1 Fig.
(EPS)

S6 Fig Histogram distributions and correlations between traits at the harvest stage in the field. Details as for S1 Fig.
(EPS)

S7 Fig Relative rank in height for RILs and parents at the three developmental stages. Each RIL is represented by a different colored solid line, and the parents with magenta (*S*. *viridis*) and blue (*S*. *italica*) dashed bolded lines.
(EPS)

S1 Table Non-parametric correlations between total biomass and plant architectural traits at the three developmental stages. Entries are Spearman rho values. Significance levels are denoted by asterisks, * P < 0.05, ** P < 0.01, ** P < 0.001.
(DOCX)

S2 Table QTL positions, % phenotypic variance explained, and additive effects. (XLSX)

S3 Table Candidate genes occurring in QTL regions. (DOCX)

S4 Table Least square means for phenotypic traits. (CSV)
