# Supplementary material for: Development and Genetic Control of Plant Architecture and Biomass in the Panicoid Grass, Setaria
Source: PLoS One. 2016 Mar 17;11(3):e0151346. doi: 10.1371/journal.pone.0151346 (PMC4795695; doi:10.1371/journal.pone.0151346)
Supplement: S1 Table — Entries are Spearman rho values. Significance levels are denoted by asterisks, * P < 0.05, ** P < 0.01, ** P < 0.001. (DOCX) [file pone.0151346.s008.docx]

Supplementary Table 1. Non-parametric correlations between total biomass and plant architectural traits at the three developmental stages. Entries are Spearman rho values. Significance levels are denoted by asterisks, * P < 0.05, ** P < 0.01, ** P < 0.001.

| Trait | Total Biomass Greenhouse | Total Biomass Field |
| --- | --- | --- |
| Vegetative height | ns | + 0.31 *** |
| Vegetative tiller number | ns | ns |
| Flowering height | + 0.58 *** | + 0.74 *** |
| Flowering tiller number | + 0.19 * | ns |
| Flowering aerial branch number | ns | ns |
| Days to flowering | + 0.66 *** | ns |
| Harvest height | + 0.52 *** | + 0.79 *** |
| Harvest tallest tiller height | + 0.51 *** | + 0.73 *** |
| Harvest node number | + 0.60 *** | + 0.48 *** |
| Harvest tiller number | – 0.39 *** | ns |
| Harvest aerial branch number | – 0.42 *** | ns |
| Days to harvest | ns | ns |
