# Supplementary material for: Development and Genetic Control of Plant Architecture and Biomass in the Panicoid Grass, Setaria
Source: PLoS One. 2016 Mar 17;11(3):e0151346. doi: 10.1371/journal.pone.0151346 (PMC4795695; doi:10.1371/journal.pone.0151346)
Supplement: S3 Table — (DOCX) [file pone.0151346.s010.docx]

Supplementary Table 3. Candidate genes for QTL regions.

| QTL region | Flanking Markers | Markers at LOD Peaks | Candidate genes | Reference |
| --- | --- | --- | --- | --- |
| Br1a | p92 - UGSF431 | UGSF409  UGSF414  UGSF415  UGSF427 | ???  ???  *LATERAL SUPPRESSOR* (*LAS*)/*MONOCULM1-like* (*MOC1-like*)/Si019936m  ??? | {Greb, 2003 #43;Li, 2003 #2}  (1)/(2) |
| Br1b | UGSF472 - UGSF497 | p16  UGSF488 | *GIBBERELLIN 2-OXIDASE9 (GA2ox9)/* Si017671m  *REDUCED SHOOT BRANCHING 1 (RSB1)/*Si018160m | (3)  (4) |
| H1a | UGSF423 - UGSF472 | UGSF427  UGSF436  UGSF460 | ???  ???  *DWARF5-like (DWF5-like)*/Si016932m; | (5) |
| Bio1a | UGSF430 - UGSF465 | UGSF436  UGSF460  UGSF465 | ???  *DWARF5-like (DWF5-like)/*Si016932m  ??? | (5) |
| Br2a | UGSF160 - MPGA39 | UGSF161  UGSF165 | *KNOTTED1-like (KN1-like)/OsKN3*/Si031454m  ??? | (6), (7)/(8) |
| Br2b | c397 - UGSF232 | UGSF229 | Osa-MIR156a-like/30830659-30830678; *BRANCHED 2 (BRC2)/*Si033183m | (9); (10) |
| Br2c | UGSF283 - UGSF284 | UGSF284 | *FRIZZY PANICLE (FZP)/branched silkless1 (bd1)/*Si032771m | (11), (12)/(13) |
| H2a | UGSF219 - UGSF231 | UGSF229 | Osa-MIR156a-like /30830659-30830678; *BRANCHED 2 (BRC2)*/Si033183m | (9); (10) |
| H2b | UGSF263 - UGSF277 | UGSF265  UGSF271 | ???  *Six-rowed spike1 (Vrs1)/*Si031009m; *INCREASED NUMBER OF TILLERS (TLD1)/DWARF IN LIGHT1-like (DFL1-like)/*Si029251m | (14); (15)/(16) |
| H2c | UGSF282 - UGSF285 | UGSF285 | ??? |  |
| Bio2a | UGSF239 - UGSF259 | UGSF246 | *MADS-BOX DOMAIN PROTEIN (OsMADS8)*/Si030960m | (17); |
| Bio2b | UGSF283 - UGSF285 | UGSF285 | ??? |  |
| Br3a | UGSF675 - UGSF821 | UGSF672  UGSF821 | *ARABIDOPSIS RESPONSE REGULATOR5 (ARR5)/ A-TYPE RESPONSE REGULATOR6 (OsRR6)*/Si023483m  *OsKN2/KNOTTED1-like HOMEODOMAIN (KN1-like)*/Si023561m | (18)/(19)  (8)/(6) |
| Br3b | UGSF782 - UGSF864 | MPGD44  UGSF877 | ???  *OsPIN3b*/Si025109m (Putative auxin efflux carrier component) |  |
| H3a | UGSF675 - UGSF821 | UGSF672  UGSF671 | *ARABIDOPSIS RESPONSE REGULATOR5 (ARR5)/A-TYPE RESPONSE REGULATOR6 (OsRR6)/*Si023483m  ??? | (18)/(19) |
| Br4a | UGSF895 - UGSF961 | d3_HaeIII | *DWARF3 (DWF3)/F-BOX LEUCIN-RICH REPEAT MAX2 HOMOLOG (D3)/*Si005956m | (20) |
| Br5a | UGSF303 - UGSF329 | b188 | *corngrass1 (cg1)/*11531587-11532171; Os-MIR156b/11531659-11531795 | (21); (22) |
| Br5b | UGSF358 - b111 | UGSF364  UGSF365 | *BRASSINOSTEROID INSENSITIVE1 (AtBRI1)/*Si000117m;  *DWARF10 (D10)/carotenoid cleavage deoxygenase8 (ccd8)/MORE AXILLARY BRANCHING 4 (MAX4)/*Si004644m; *HIGH TILLERING, REDUCED HEIGHT, AND INFERTILE SPIKELETS (THIS1)/*Si001609m | (23), (24),(25)  (26)/(27)/(28); (29) |
| Br5c | UGSF368 - UGSF387 | UGSF385  UGSF386 | ???  *MADS-BOX DOMAIN PROTEIN (OsMADS57)*/*MADS-BOX TRANSCRIPTION FACTOR 21-like (AGL21-like)/*Si002551m | Q6Z6W2\|OsMADS57_ORYSJ This one is not the one from Guo etal 2013 “Interact between OsmADS57 and OsTB1) |
| Br5d | p2 - UGSF391 | UGSF389 | *terminal ear1 (te1*)/*LEAFY HEAD 2 (LHD2)/*Si004206m | (30)/(31) |
| H5a | UGSF299 - UGSF341 | UGSF329  UGSF335 | *SHOOTLESS 2 (SHL2)*  ??? | (32) |
| H5b | UGSF367 - UGSF388 | UGSF384  UGSF385 | ???  *GIBBERELLIN 20 OXIDASE-like (GA20OX-like)/*Si001573m | (33) |
| H5c | p2 - b223 | UGSF389 | *terminal ear1 (te1*)// *LEAFY HEAD 2 (LHD2)*/Si004206m | (30)/(31) |
| Bio5a | UGSF304 - UGSF299 | UGSF300 | *GLUTAMINE AMIDOTRANSFERASE (GAT)/*Si001508m | (34) |
| Bio5b | b188 - UGSF350 | UGSF335 | ??? |  |
| Bio5c | UGSF350 - UGSF365 | UGSF364 | *BRASSINOSTEROID INSENSITIVE1 (AtBRI1)/*Si000117m | (23), (24),(25) |
| Br6a | UGSF691 - UGSF745 | UGSF686 | ??? |  |
| H6a | UGSF692 - UGSF683 | UGSF688  UGSF687 | ???  ??? |  |
| Br7a | UGSF621 - UGSF626 | UGSF622  UGSF626 | ???  *ISOPENTENYL TRANSFERASE2 (IPT2)/*Si012132m | (35) |
| Br7b | UGSF668 - b200 | b107  b200 | *GIBBERELLIN 2-OXIDASE (GA2OX1)/*Si012495m;  ??? | (36), (37) |
| H7a | UGSF626 - UGSF633 | g271, UGSF630 | ??? |  |
| H7b | UGSF640 - UGSF650 | UGSF646 | Os-MIR156a-like/24139286-24139305; *gibberellin 2-beta-dioxygenase (ga2ox7)/*Si012266m | (9) |
| H7c | UGSF779 - UGSF777 | b107  b200 | *GIBBERELLIN 2-OXIDASE (GA2OX1)*/Si012495m; *gibberellin 2-beta-dioxygenase (ga2ox)/*/Si012158m  ??? | (36), (37) |
| Bio7a | UGSF666 - UGSF777 | b107 | *GIBBERELLIN 2-OXIDASE (GA2OX1)*/Si012495m; *gibberellin 2-beta-dioxygenase (ga2ox)/*Si012158m | (36), (37) |
| H8a | UGSF507 - UGSF522 | UGSF509  UGSF513 | *TWISTED DWARF 1 (TWD1)/*Si026525m; *terminal flower1 (tfl1)/Terminal flower1-like (Tfl1-like)/*Si028168m  ??? | (38)/(39),(40) |
| Bio8a | UGSF507 - UGSF526 | UGSF513 | ??? |  |
| Br9a | p44 - UGSF32 | b246  UGSF23  UGSF25 | *SHOOT MERISTEMLESS (STM)/*Si036193m; *knotted1 (kn1)/*Si038700m; *knotted1 (kn1)/*Si037920m  *Phytochrome A (PHYA)/phytochrome A (PhyA2)*/Si033984m  *teosinte branched (tb1)/*Si038692m | (41),(42); (43),(44)  (45),(46)  (47), (48) |
| Br9b | MPGA41 - UGSF128 | UGSF121  UGSF125 | ???  *Phytochrome B (PHYB)/phytochrome B (PhyB1)/*Si033968m | (49) |
| Br9c | UGSF129 - UGSF149 | UGSF132  p41  UGSF145 | *REDUCED CULM NUMBER1-like (Rcn1-like)/*Si040245m; *REDUCED CULM NUMBER1 (Rcn1)/*Si039677m  *SHADE AVOIDANCE 1 (ATDWF4)/ DWARF4 (D4)/*Si035274m  *DWARF88 (D88)/DWARF14 (D14)/*Si036746m; *grassy tillers1 (gt1)*/Si037255m | (50)  (51), (52)/(53)  (54), (55); (56) |
| H9a | p44 - UGSF31 | UGSF19  p4  UGSF23 | *PHYTOCHROME C (PHYC)/Phytochrome C (PhyC)/*Si034030m; *MADS-BOX DOMAIN PROTEIN (OsMADS14)/* Si037627m  *SHOOT MERISTEMLESS (STM)/*Si036193m; *knotted1 (kn1)/*Si038700m; *knotted1 (kn1)/*Si037920m  *Phytochrome A (PHYA)/phytochrome A (PhyA2)*/Si033984m; ZmDWARF8/Si039400m | (57), (58); (17)  (41),(42); (43),(44)  (45),(46); (59) |
| H9b | UGSF112 - UGSF121 | UGSF119  UGSF121 | ???  ??? |  |
| Bio9a | UGSF12 - UGSF29 | UGSF19  p4 | *PHYTOCHROME C (PHYC)/Phytochrome C (PhyC)/*Si034030m; *MADS-BOX DOMAIN PROTEIN (OsMADS14)/* Si037627m  *SHOOT MERISTEMLESS (STM)/*Si036193m; *knotted1 (kn1)/*Si038700m; *knotted1 (kn1)/*Si037920m | (57), (58); (17)  (41),(42); (43),(44) |

References

1. Greb T, Clarenz O, Schafer E, Muller D, Herrero R, Schmitz G, et al. Molecular analysis of the LATERAL SUPPRESSOR gene in Arabidopsis reveals a conserved control mechanism for axillary meristem formation. Genes & Development. 2003;17(9):1175-87.

2. Li XY, Qian Q, Fu ZM, Wang YH, Xiong GS, Zeng DL, et al. Control of tillering in rice. Nature. 2003;422(6932):618-21.

3. Lo SF, Yang SY, Chen KT, Hsing YL, Zeevaart JAD, Chen LJ, et al. A Novel Class of Gibberellin 2-Oxidases Control Semidwarfism, Tillering, and Root Development in Rice. Plant Cell. 2008;20(10):2603-18.

4. Huang X, Effgen S, Meyer RC, Theres K, Koornneef M. Epistatic Natural Allelic Variation Reveals a Function of AGAMOUS-LIKE6 in Axillary Bud Formation in Arabidopsis. The Plant Cell. 2012;24(6):2364-79.

5. Choe S, Tanaka A, Noguchi T, Fujioka S, Takatsuto S, Ross AS, et al. Lesions in the sterol Delta(7) reductase gene of Arabidopsis cause dwarfism due to a block in brassinosteroid biosynthesis. Plant Journal. 2000;21(5):431-43.

6. Vollbrecht E, Reiser L, Hake S. Shoot meristem size is dependent on inbred background and presence of the maize homeobox gene, knotted1. Development. 2000;127(14):3161-72.

7. Bolduc N, Hake S. The Maize Transcription Factor KNOTTED1 Directly Regulates the Gibberellin Catabolism Gene ga2ox1. Plant Cell. 2009;21(6):1647-58.

8. Postma-Haarsma AD, Rueb S, Scarpella E, den Besten W, Hoge JH, Meijer AH. Developmental regulation and downstream effects of the knox class homeobox genes Oskn2 and Oskn3 from rice. Plant Mol Biol. 2002;48(4):423-41.

9. Luo L, Li WQ, Miura K, Ashikari M, Kyozuka J. Control of Tiller Growth of Rice by OsSPL14 and Strigolactones, Which Work in Two Independent Pathways. Plant and Cell Physiology. 2012;53(10):1793-801.

10. Aguilar-Martinez JA, Poza-Carrion C, Cubas P. Arabidopsis BRANCHED1 acts as an integrator of branching signals within axillary buds. Plant Cell. 2007;19(2):458-72.

11. Komatsu M, Maekawa M, Shimamoto K, Kyozuka J. The LAX1 and FRIZZY PANICLE 2 genes determine the inflorescence architecture of rice by controlling rachis-branch and spikelet development. Developmental Biology. 2001;231(2):364-73.

12. Komatsu M, Chujo A, Nagato Y, Shimamoto K, Kyozuka J. FRIZZY PANICLE is required to prevent the formation of axillary meristems and to establish floral meristem identity in rice spikelets. Development. 2003;130(16):3841-50.

13. Colombo L, Marziani G, Masiero S, Wittich PE, Schmidt RJ, Gorla MS, et al. BRANCHED SILKLESSmediates the transition from spikelet to floral meristem duringZea maysear development. The Plant Journal. 1998;16(3):355-63.

14. Komatsuda T, Pourkheirandish M, He C, Azhaguvel P, Kanamori H, Perovic D, et al. Six-rowed barley originated from a mutation in a homeodomain-leucine zipper I-class homeobox gene. Proc Natl Acad Sci U S A. 2007;104(4):1424-9.

15. Zhang SW, Li CH, Cao J, Zhang YC, Zhang SQ, Xia YF, et al. Altered architecture and enhanced drought tolerance in rice via the down-regulation of indole-3-acetic acid by TLD1/OsGH3.13 activation. Plant Physiol. 2009;151(4):1889-901.

16. Hagen G, Guilfoyle T. Auxin-responsive gene expression: genes, promoters and regulatory factors. Plant Mol Biol. 2002;49(3-4):373-85.

17. Jeon JS, Lee S, Jung KH, Yang WS, Yi GH, Oh BG, et al. Production of transgenic rice plants showing reduced heading date and plant height by ectopic expression of rice MADS-box genes. Molecular Breeding. 2000;6(6):581-92.

18. D'Agostino IB, Deruere J, Kieber JJ. Characterization of the response of the Arabidopsis response regulator gene family to cytokinin. Plant Physiol. 2000;124(4):1706-17.

19. Hirose N, Makita N, Kojima M, Kamada-Nobusada T, Sakakibara H. Overexpression of a type-A response regulator alters rice morphology and cytokinin metabolism. Plant Cell Physiol. 2007;48(3):523-39.

20. Zhao J, Wang T, Wang M, Liu Y, Yuan S, Gao Y, et al. DWARF3 participates in an SCF complex and associates with DWARF14 to suppress rice shoot branching. Plant Cell Physiol. 2014;55(6):1096-109.

21. Chuck G, Cigan AM, Saeteurn K, Hake S. The heterochronic maize mutant Corngrass1 results from overexpression of a tandem microRNA. Nat Genet. 2007;39(4):544-9.

22. Xie K, Wu C, Xiong L. Genomic organization, differential expression, and interaction of SQUAMOSA promoter-binding-like transcription factors and microRNA156 in rice. Plant physiology. 2006;142(1):280-93.

23. Yamamuro C, Ihara Y, Wu X, Noguchi T, Fujioka S, Takatsuto S, et al. Loss of function of a rice brassinosteroid insensitive1 homolog prevents internode elongation and bending of the lamina joint. The Plant Cell. 2000;12(9):1591-605.

24. Friedrichsen DM, Joazeiro CA, Li J, Hunter T, Chory J. Brassinosteroid-insensitive-1 is a ubiquitously expressed leucine-rich repeat receptor serine/threonine kinase. Plant Physiol. 2000;123(4):1247-56.

25. Wang ZY, Seto H, Fujioka S, Yoshida S, Chory J. BRI1 is a critical component of a plasma-membrane receptor for plant steroids (vol 410, pg 380, 2001). Nature. 2001;411(6834):219-.

26. Arite T, Iwata H, Ohshima K, Maekawa M, Nakajima M, Kojima M, et al. DWARF10, an RMS1/MAX4/DAD1 ortholog, controls lateral bud outgrowth in rice. Plant J. 2007;51(6):1019-29.

27. Guan JC, Koch KE, Suzuki M, Wu S, Latshaw S, Petruff T, et al. Diverse roles of strigolactone signaling in maize architecture and the uncoupling of a branching-specific subnetwork. Plant Physiol. 2012;160(3):1303-17.

28. Sorefan K, Booker J, Haurogne K, Goussot M, Bainbridge K, Foo E, et al. MAX4 and RMS1 are orthologous dioxygenase-like genes that regulate shoot branching in Arabidopsis and pea. Genes Dev. 2003;17(12):1469-74.

29. Liu W, Zhang DC, Tang MF, Li DY, Zhu YX, Zhu LH, et al. THIS1 is a putative lipase that regulates tillering, plant height, and spikelet fertility in rice. Journal of Experimental Botany. 2013;64(14):4389-402.

30. Veit B, Briggs SP, Schmidt RJ, Yanofsky MF, Hake S. Regulation of leaf initiation by the terminal ear 1 gene of maize. Nature. 1998;393(6681):166-8.

31. Xiong GS, Hu XM, Jiao YQ, Yu YC, Chu CC, Li JY, et al. LEAFY HEAD2, which encodes a putative RNA-binding protein, regulates shoot development of rice. Cell Research. 2006;16(3):267-76.

32. Nagasaki H, Itoh JI, Hayashi K, Hibara KI, Satoh-Nagasawa N, Nosaka M, et al. The small interfering RNA production pathway is required for shoot meristern initiation in rice. Proceedings of the National Academy of Sciences of the United States of America. 2007;104(37):14867-71.

33. Kende H, Zeevaart J. The Five" Classical" Plant Hormones. The plant cell. 1997;9(7):1197.

34. Zhu H, Kranz RG. A Nitrogen-Regulated Glutamine Amidotransferase (GAT1_2.1) Represses Shoot Branching in Arabidopsis. Plant Physiology. 2012;160(4):1770-80.

35. Sakamoto T, Sakakibara H, Kojima M, Yamamoto Y, Nagasaki H, Inukai Y, et al. Ectopic expression of KNOTTED1-like homeobox protein induces expression of cytokinin biosynthesis genes in rice. Plant Physiology. 2006;142(1):54-62.

36. Sakamoto T, Kobayashi M, Itoh H, Tagiri A, Kayano T, Tanaka H, et al. Expression of a gibberellin 2-oxidase gene around the shoot apex is related to phase transition in rice. Plant Physiology. 2001;125(3):1508-16.

37. Sakamoto T, Morinaka Y, Ishiyama K, Kobayashi M, Itoh H, Kayano T, et al. Genetic manipulation of gibberellin metabolism in transgenic rice. Nature Biotechnology. 2003;21(8):909-13.

38. Geisler M, Kolukisaoglu HU, Bouchard R, Billion K, Berger J, Saal B, et al. TWISTED DWARF1, a unique plasma membrane-anchored immunophilin-like protein, interacts with Arabidopsis multidrug resistance-like transporters AtPGP1 and AtPGP19. Molecular Biology of the Cell. 2003;14(10):4238-49.

39. Shannon S, Meeks-Wagner DR. A mutation in the Arabidopsis TFL1 gene affects inflorescence meristem development. The Plant Cell. 1991;3(9):877-92.

40. Chang LL, Wu LC, Chen YH, Ku LX, Yang S, Zhang SF, et al. Expression and Functional Analysis of the ZCN1(ZmTFL1) Gene, a TERMINAL FLOWER 1 Homologue that Regulates the Vegetative to Reproductive Transition in Maize. Plant Molecular Biology Reporter. 2012;30(1):55-66.

41. Barton MK, Poethig RS. Formation of the Shoot Apical Meristem in Arabidopsis-Thaliana - an Analysis of Development in the Wild-Type and in the Shoot Meristemless Mutant. Development. 1993;119(3):823-31.

42. Long JA, Moan EI, Medford JI, Barton MK. A member of the KNOTTED class of homeodomain proteins encoded by the STM gene of Arabidopsis. Nature. 1996;379(6560):66-9.

43. Vollbrecht E, Veit B, Sinha N, Hake S. The Developmental Gene Knotted-1 Is a Member of a Maize Homeobox Gene Family. Nature. 1991;350(6315):241-3.

44. Jackson D, Veit B, Hake S. Expression of Maize Knotted1 Related Homeobox Genes in the Shoot Apical Meristem Predicts Patterns of Morphogenesis in the Vegetative Shoot. Development. 1994;120(2):405-13.

45. Foster KR, Miller FR, Childs KL, Morgan PW. Genetic-Regulation of Development in Sorghum-Bicolor .7. Shoot Growth, Tillering, Flowering, Gibberellin Biosynthesis, and Phytochrome Levels Are Differentially Affected by Dosage of the Ma(3)(R) Allele. Plant Physiology. 1994;105(3):941-8.

46. Kong SG, Lee DS, Kwak SN, Kim JK, Sohn JK, Kim IS. Characterization of sunlight-grown transgenic rice plants expressing Arabidopsis phytochrome A. Molecular Breeding. 2004;14(1):35-45.

47. Doebley J, Stec A, Gustus C. Teosinte Branched1 and the Origin of Maize - Evidence for Epistasis and the Evolution of Dominance. Genetics. 1995;141(1):333-46.

48. Doebley J, Stec A, Hubbard L. The evolution of apical dominance in maize. Nature. 1997;386(6624):485-8.

49. Kebrom TH, Burson BL, Finlayson SA. Phytochrome B represses Teosinte Branched1 expression and induces sorghum axillary bud outgrowth in response to light signals. Plant Physiology. 2006;140(3):1109-17.

50. Yasuno N, Takamure I, Kidou S-i, Tokuji Y, Ureshi A-n, Funabiki A, et al. Rice shoot branching requires an ATP-binding cassette subfamily G protein. New Phytologist. 2009;182(1):91-101.

51. Choe SW, Dilkes BP, Fujioka S, Takatsuto S, Sakurai A, Feldmann KA. The DWF4 gene of Arabidopsis encodes a cytochrome P450 that mediates multiple 22 alpha-hydroxylation steps in brassinosteroid biosynthesis. Plant Cell. 1998;10(2):231-43.

52. Choe S, Fujioka S, Noguchi T, Takatsuto S, Yoshida S, Feldmann KA. Overexpression of DWARF4 in the brassinosteroid biosynthetic pathway results in increased vegetative growth and seed yield in Arabidopsis. Plant Journal. 2001;26(6):573-82.

53. Sakamoto T, Morinaka Y, Ohnishi T, Sunohara H, Fujioka S, Ueguchi-Tanaka M, et al. Erect leaves caused by brassinosteroid deficiency increase biomass production and grain yield in rice. Nat Biotech. 2006;24(1):105-9.

54. Kinoshita T, Takahashi M-e. The one hundredth report of genetical studies on rice plant: Linkage studies and future prospects. Journal of the Faculty of Agriculture, Hokkaido University= 北海道大學農學部紀要. 1991;65(1):1-61.

55. Ishikawa S, Maekawa M, Arite T, Onishi K, Takamure I, Kyozuka J. Suppression of tiller bud activity in tillering dwarf mutants of rice. Plant and Cell Physiology. 2005;46(1):79-86.

56. Whipple CJ, Kebrom TH, Weber AL, Yang F, Hall D, Meeley R, et al. grassy tillers1 promotes apical dominance in maize and responds to shade signals in the grasses. Proceedings of the National Academy of Sciences of the United States of America. 2011;108(33):E506-E12.

57. Basu D, Dehesh K, Schneider-Poetsch HJ, Harrington SE, McCouch SR, Quail PH. Rice PHYC gene: structure, expression, map position and evolution. Plant Molecular Biology. 2000;44(1):27-42.

58. Childs KL, Miller FR, CordonnierPratt MM, Pratt LH, Morgan PW, Mullet JE. The sorghum photoperiod sensitivity gene, Ma(3), encodes a phytochrome B. Plant Physiology. 1997;113(2):611-9.

59. Thornsberry JM, Goodman MM, Doebley J, Kresovich S, Nielsen D, Buckler ES. Dwarf8 polymorphisms associate with variation in flowering time. Nature Genetics. 2001;28(3):286-9.
